# Recombinant human growth hormone (rhGH) treatment of MKN-45 xenograft mice improves nutrition status and strengthens immune function without promoting tumor growth

**DOI:** 10.1371/journal.pone.0210613

**Published:** 2019-01-23

**Authors:** Lianping Wei, Jianrong Chang, Zhen Han, Ronghai Wang, Lihua Song

**Affiliations:** 1 College of Life Science, Anhui Agricultural University, Hefei, Anhui Province, People’s Republic of China; 2 Scientific research center, Benbu Medical college, Benbu, Anhui Province, People’s Republic of China; 3 AnHui Anke Biotechnology (Group) Co.,Ltd. Hefei, Anhui Province, People’s Republic of China; University of South Alabama Mitchell Cancer Institute, UNITED STATES

## Abstract

The aim of this study was to clarify the combined effects and dose-effect relationships of rhGH on tumor growth, nutrition status, and immune function in MKN-45 xenograft mice. In this study, animal models were induced in nude mice using the subcutaneous transplantation of MKN-45 cells, and rhGH was injected daily for 14 days. Three rhGH treatment dosages were set with reference to the equivalent dosage converted from human clinical dosage, including 2 IU (0.67 mg), 10 IU (3.35 mg) and 50 IU (16.75 mg) per kg body weight. The tumor volume, body weight and food intake were measured every two or three days. After 14 days of rhGH treatment, the tumors were isolated and weighed. The expression levels of Ki-67, vascular endothelial growth factor (VEGF) and CD31in tumor tissues were detected by immunohistochemistry (IHC). The protein expression levels of pJAK2, JAK2, pSTAT3, STAT3, pAKT, AKT, pERK and ERK were measured by western blotting. The percentage of active NK cells in peripheral blood mononuclear cells (PBMCs) was detected by fluorescence-activated cell sorting (FACS). The results showed that rhGH had improved the food intake, increased the body weight and strengthened the immune function of MKN-45 xenograft mice but had not promote tumor growth. MKN-45 xenograft mice treated with rhGH at a higher dosage gained more weight, while those treated with rhGH at a lower dosage showed stronger immune function and smaller tumor volume.

## Introduction

Cancer cachexia is a complex metabolic syndrome characterized by loss of body weight, reduced food intake and severe malnutrition[1–3]. Additionally, this syndrome is always associated with impaired physical function, poor responsiveness to antineoplastic therapies, decreased quality of life and increased rates of morbidity and mortality[4–5]. Approximately 60% to 80% of patients with advanced cancer suffer from cancer cachexia, and this ratio is higher in patients with gastric cancer[6–7]. However, conventional anticachectic therapy with nutritional support is not efficient Therefore, it is important for patients with gastric cancer to identify new ways to overcome cancer cachexia[8–9].

Growth hormone (GH) is a 191-amino acid peptide naturally released by the anterior pituitary gland that plays a significant role in the regulation of substrate metabolism and body composition in human[10]. GH is a potent anabolic agent that can reverse many nutritional and metabolic abnormalities associated with severe catabolic states[11–12]. Similar to GH, recombinant human GH (rhGH), which is produced by recombinant DNA technology, has been shown to stimulate muscle protein synthesis, improve nitrogen balance, promote wound healing and strengthen the immune system. Currently, rhGH has been approved by the US Food and Drug Administration for use in HIV/AIDS wasting and parenteral nutrition-dependent short-bowel syndrome [13]. However, rhGH is not used in patients with advanced cancers because rhGH has been associated with an increased risk of cancer[14–18]. As a mitogen, rhGH may promote cell renewal and increase malignant transformation by binding to GH receptor (GHR) on the tumor cell surface, resulting in the activation of various signaling pathways[19–22]. Therefore, it is necessary to evaluate the risks and benefits of rhGH treatment in patients with gastric cancer.

Research results concerning whether rhGH promotes cancer cell proliferation or tumor growth are inconsistent. Some reports have indicated that rhGH does not promote the proliferation of cancer cells [11,23]. The causes of these inconsistent results may be multifactorial. The level of GHR expression on the tumor cell membrane surface has been considered to play a key role in tumor growth induced by rhGH[24]. Animal experiments have confirmed that rhGH does not promote the growth of MKN-45 xenograft tumors in vivo, in which GHR is negatively expressed. The authors have indicated that rhGH treatment may be safe for cancer patients with no GHR expression in tumors[24–25]. However, in these studies, the beneficial effect of rhGH on nutrition status and immune function has not been investigated. Nutritional status, immune function and tumor growth are interdependent, making it necessary to assess the combined effects of GH on tumor-bearing mice. In addition, the dosage of rhGH treatment also affects the risks and benefits of rhGH treatment. The dosages of rhGH in the previous study were much greater than the equivalent dosage converted from the human clinical dosage according to body surface area. RhGH treatment with dosages equivalent to or lower than the clinical dosage has not been studied. Therefore, the study of the dose-effect relationships of rhGH on tumor growth, nutritional status and immunity has not been comprehensive.

The aim of this study was to clarify the combined effects of rhGH on tumor growth, nutrition status, and immune function in MKN-45 tumor-bearing mice. At the same time, the dose-effect relationships were comprehensively investigated. Based on these results, we assessed the benefits and risks of rhGH treatment in MKN-45 tumor-bearing mice.

## Materials and methods

### Cell lines and culture

Cells of the human gastric cancer cell lines SGC-7901, MGC-803 and MKN-45 were purchased from the Chinese Academy of Medical Science. The cells were cultured in RPMI 1640 (HyClone Laboratories, Logan, USA) supplemented with 10% fetal bovine serum (FBS; HyClone Laboratories, Logan, USA) and 1% penicillin-streptomycin solution (HyClone Laboratories, Logan, USA). The cells were incubated under humidified conditions at 37°C and 5% CO_2_.

### Animal experiment

Female BALB/c nude mice (SPF, weight: 16–18 g) were purchased from Beijing Vital River Laboratory Animal Technology Co., Ltd. (Beijing, China; SCXK2012-0001). All mice were maintained with sterilized food and water and were housed at six mice per cage in standard polycarbonate cages with controlled temperature and humidity and a 12-h light and dark cycle. Mice were allowed to acclimate to laboratory conditions for 7 days prior to experimentation. All procedures were carried out in strict accordance with the recommendations in the Guide for the Care and Use of Laboratory Animals of Anhui Anke Biotechnology (Group) Co.,Ltd. These guidelines met the ethical standards concerning experimental animals in China, as required by law. The protocol was approved by the Committee on the Ethics of Animal Experiments of Anhui Anke Biotechnology (Group) Co.,Ltd. (Protocol Number: AK-20151225). All efforts were made to minimize suffering.

### Nude mouse xenograft tumor assay

MKN-45 cells (1×10^7^in100μLof medium) were inoculated subcutaneously into the right front armpit of nude mice. When the tumor volumes reached approximately 100-200mm^3^, the mice were randomized into four groups with six mice per group: the low-dosage group received 2 IU/kg (0.67 mg/kg) body weight rhGH (Anhui Anke Biotechnology (Group) Co., Ltd. Anhui, China) daily; the middle-dosage group received 10 IU/kg (3.35 mg/kg) body weight rhGH daily; the high-dosage group received 50 IU/kg (16.75 mg/kg) body weight rhGH daily; and the control group received normal saline daily. The tumor volume, body weight and food intake were measured every two or three days. The tumor volumes were determined using the following formula: volume = length×width^2^×0.5. After 14 days, the mice were anesthetized by inhalation of 1.0–2.5% isoflurane, and blood was collected from the heart. Anesthetized mice were sacrificed by carbon dioxide asphyxiation. The tumors were isolated, weighed and immediately frozen in liquid nitrogen. The tumor-free body weight at the last time point was calculated using the following formula: tumor-free body weight = (body weight with tumor)–(tumor weight).

### HE staining and IHC assay for tumor tissues

After tumor tissues were isolated from mice, they were fixed in 4% paraformaldehyde, embedded in paraffin and cut into 4-μmsections. The sections were then paraffinized and stained with hematoxylin and eosin (HE). For immunohistochemistry (IHC) analysis, the tumor sections (4μm thick) were blocked and incubated with antibodies against Ki-67 (Abcam, Massachusetts, USA), vascular endothelial growth factor (VEGF:Boster Biological Company of China, Wuhan, China) and CD31(Boster Biological Company of China, Wuhan, China) overnight at 4°C. Subsequently, immunostaining was performed according to the standard protocol of the DAB Substrate Kit (ZhongshanJinqiao Corp. Beijing, China). The sections were counterstained with HE and analyzed under a Nikon80i fluorescence microscope(Tokyo, Japan). Positive cells were stained brown in the membrane or cytoplasm. The percentage of positive cells was quantified using the JEOR 801D morphological image analysis system (Version 6.0).

### Western blotting

The expression levels of GHR and in SGC-7901, MGC-803 and MKN-45 cell lines and those of the JAK2, pJAK2, STAT3, pSTAT3, ERK, pERK, AKT and pAKT proteins in control cells or rhGH-treated cells were measured by western blotting. The method of western blotting is carried out according to Jiang et al[26]. The relative optical densities of the bands were quantified using Quantity One software. All western blot analyses were carried out at least three times.

### FACS analysis of NK cell activity

The NK cell activity of peripheral blood mononuclear cells(PBMCs) in MKN-45 xenograft mice was detected by fluorescence-activated cell sorting (FACS). At the end of the experiment, the mice were sacrificed, and peripheral blood was collected. The red blood cells were removed from the peripheral blood using a whole-blood red cell lysing reagent, and the PBMCs were collected. The PBMCs were incubated with specific fluorochrome-conjugated monoclonal antibodies, including anti-mouse CD314 APC (eBioscience, CA, USA) and anti-mouse pan-NK Cells PE (eBioscience, CA, USA), for 30min in the dark at 4°C. The percentage of active NK cells of PBMCs was assessed by flow cytometry (Beckman FC 500, CA, USA) and analyzed using Flow Jo Software (version 7.6).

### Statistical analysis

The data are shown as the mean±SEM. The statistical significance of the difference between the control and rhGH-treatment groups was determined by Student’s t-test. Values of *p* < 0.05 were considered statistically significant.

## Results

### Effect of rhGH on tumor growth in MKN-45 xenograft mice

**Fig 1:**
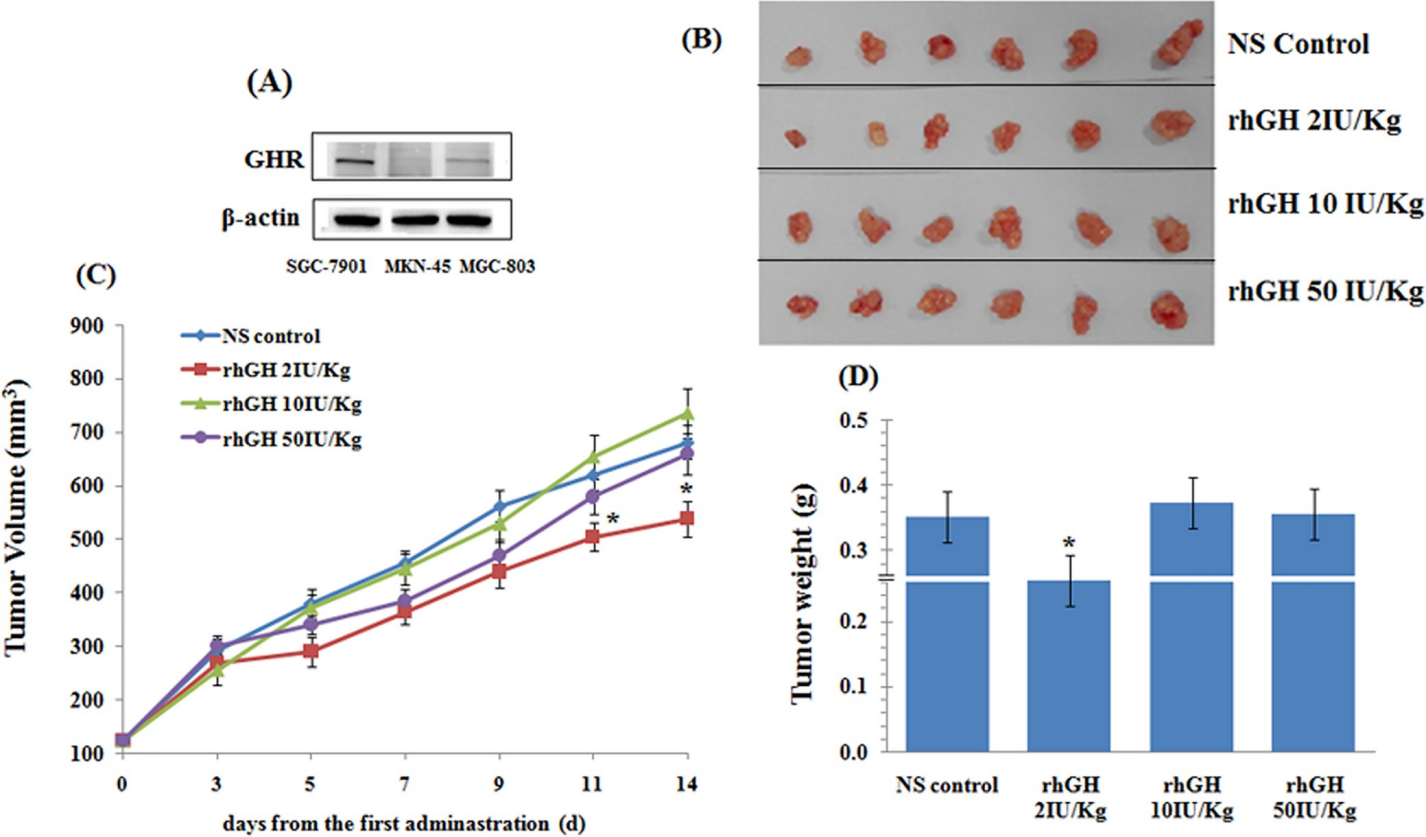
Effect of rhGH on the growth of MKN-45 xenograft tumors. (A) GHR expression in three gastric carcinoma cell lines. (B) Representative pictures of tumors in each group. (C) Tumor volume growth curve in each group. (D) Tumor weight in each group. The tumor volumes were measured in each group at the indicated time points with various treatments. The data are presented as the mean± SEM (n = 6 mice/group). *p< 0.05, treatment groups versus the NS control.

As shown in Fig 1A, the lanes of the SGC-7901 and MGC-803 cell lines have clear protein bands, while the lane of the MKN-45 cell line has no protein band. It showed that the MKN-45 cell line is a human gastric carcinoma cell line in which GHR is negatively expressed. To clarify the effect of rhGH on MKN-45 xenograft tumor growth in vivo, rhGH was administered at 2, 10 and 50 IU/kg body weight to MKN-45 xenograft mice daily for 14 days by intraperitoneal injection, and normal saline in the absence of rhGH was injected as the negative control. At the end of the experiment, the tumors were isolated and weighed (Fig 1B). As shown in Fig 1C and 1D, MKN-45 xenograft tumors progressively grew in the control group. No obvious difference was noted in the tumor volume and weight between the rhGH high-dosage group or rhGH middle-dosage group and the control group. However, the tumor volume and weight both significantly decreased (*p*<0.05) in the rhGH low-dosage group compared with those in the control group. These data suggest that rhGH did not promote MKN-45 xenograft tumor growth in vivo and that rhGH at a low dosage suppressed the growth of MKN-45 xenograft tumors.

### Effect of rhGH on cell proliferation and angiogenesis in MKN-45 xenograft tumors

**Fig 2:**
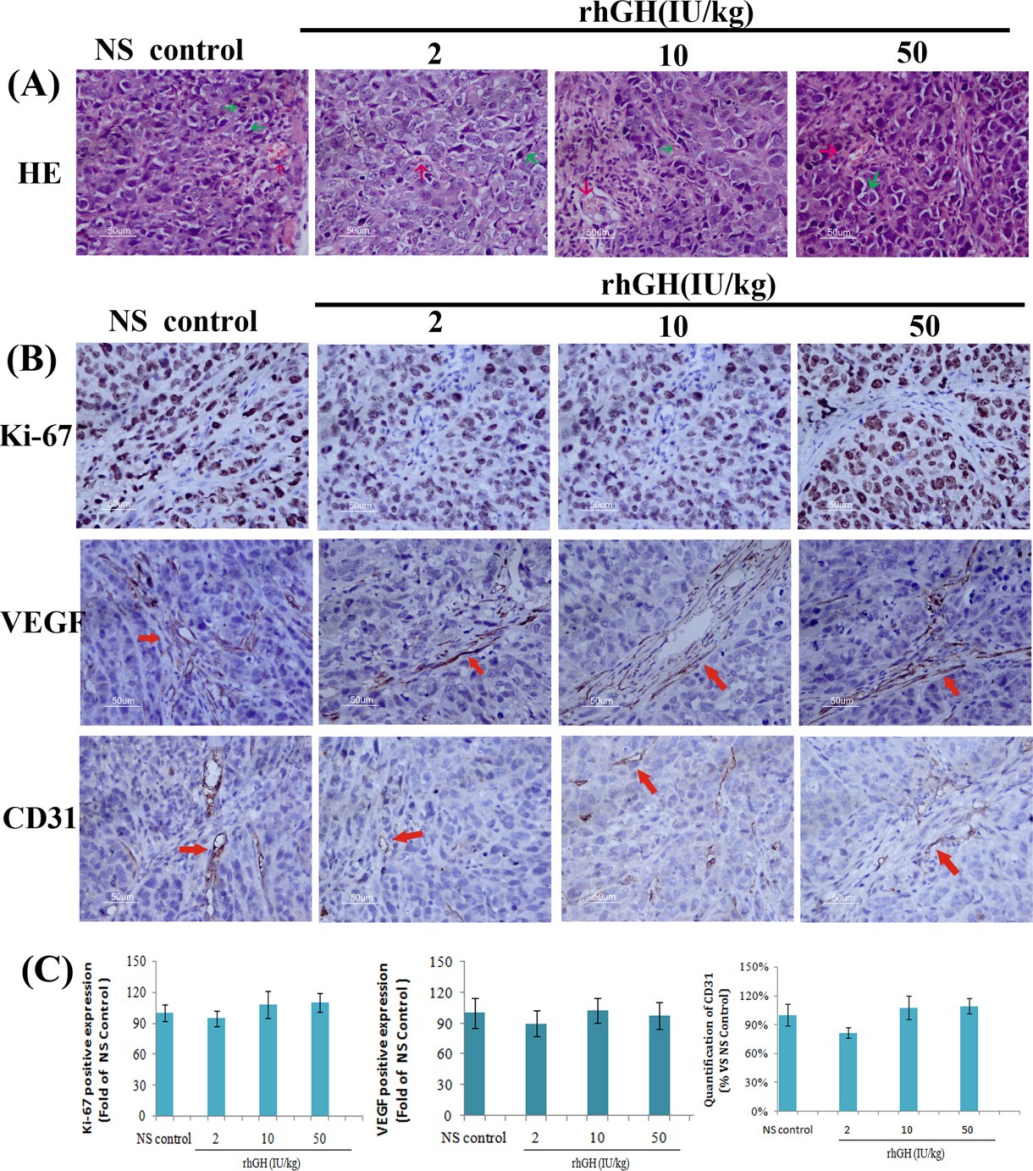
HE and IHC analysis of MKN-45 xenograft tumors. (A) Representative photomicrographs of MKN-45 xenograft tumor sections stained by HE (200× original magnifications: green arrows represent the mitoses of tumor cells; red arrows represent blood vessels inside the tumor). (B) Representative photomicrographs of Ki-67, VEGF and CD31 expression in MKN-45 xenograft tumor sections (200×original magnification; red arrows represent VEGF-positive expression; red arrows represent CD31-positive expression) (C) Quantification of Ki-67, VEGF and CD31 expression in tumor tissue. The data are expressed as a histogram of the mean ±SEM of three independent experiments.

MKN-45 xenograft tumor sections stained with HE (Fig 2A) showed the mitoses of tumor cells and rich blood vessels inside the tumor. Uncontrolled tumor cell proliferation and angiogenesis are characteristic features of most cancers. To further evaluate the rhGH-mediated effect on cell proliferation and angiogenesis in MKN-45 xenograft tumors, we conducted IHC to detect the expression of the cell proliferation marker Ki-67 and angiogenes is markers VEGF and CD31 in MKN-45 tumor xenograft tumors (Fig 2B). As shown in Fig 2C, the expression levels of Ki-67, VEGF and CD31 showed no significant difference between the rhGH high-dosage group or rhGH middle-dosage group and control group but were decreased in the rhGH low-dosage group, a finding in accordance with the rhGH-mediated effect on MKN-45 xenograft tumor growth. However, the decrease was not statistically significant.

### Effect of rhGH on GHR-related pathways in MKN-45 xenograft mice

**Fig 3:**
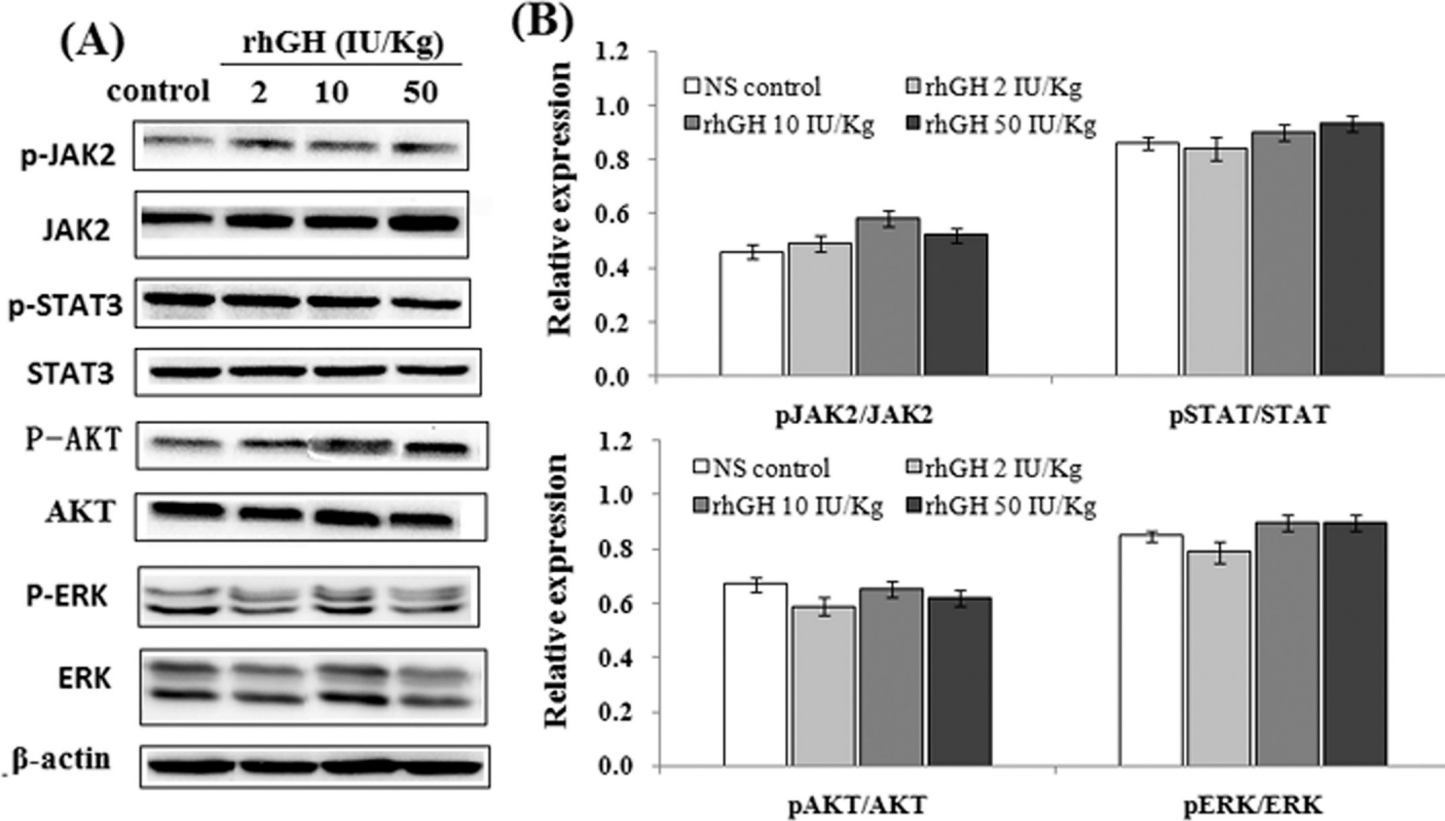
Effect of rhGH on GHR-related pathways in MKN-45 xenograft mice. (A) Western blot analysis of p-JAK2, JAK2, p-STAT3, STAT3, p-AKT, AKT, p-ERK and ERK in MKN-45 xenograft tumor tissues. (B) Quantitative analysis of the relative expression. The data are expressed as a histogram of the mean ± SEM of three independent experiments.

To further confirm whether rhGH can activate GHR-related pathways in vivo, MKN-45 xenograft tumor tissues were homogenized, lysed and analyzed by western blotting (Fig 3A). Similar to our invitro results, no significant differences were found in the relative expression of pJAK2/JAK2, pSTAT/STAT, pAKT/AKT and pERK/ERK between the rhGH-treated groups and control group (Fig 3B), suggesting that rhGH can not activate GHR-related pathways in MKN-45 xenograft mice, including the pJAK2-STAT, MAPK-ERK and AKT-PI3K signaling pathways.

### Effect of rhGH on food intake and body weight in MKN-45 xenograft mice

**Fig 4:**
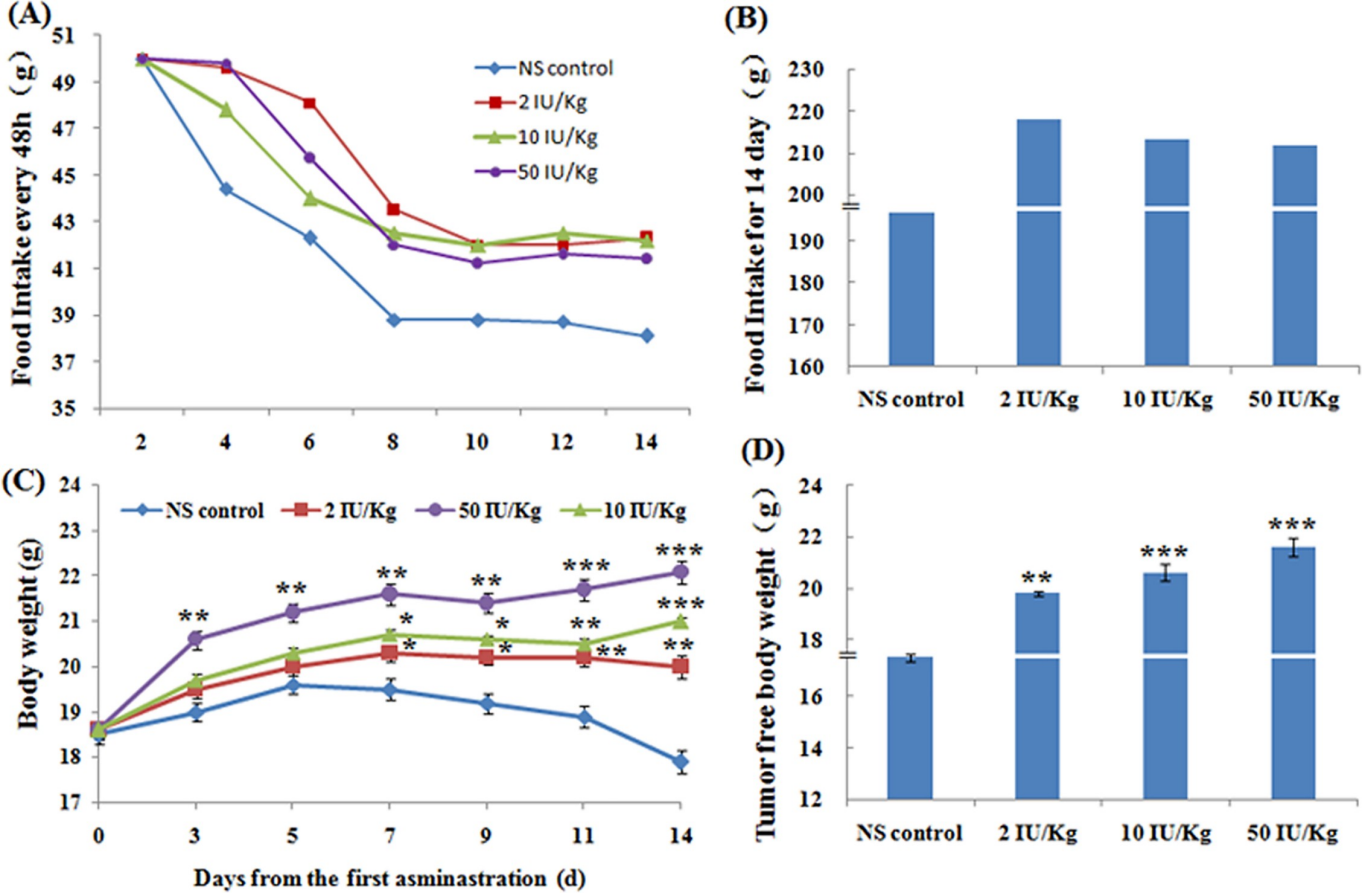
Effect of rhGH on food intake and body weight in MKN-45 xenograft mice. (A) Curve of food intake every 48 h in each group. (B) Food intake for 14 days in each group after the first rhGH administration. (C) Curve of the body weight in each group. (D) Tumor-free body weight in each group. The data are expressed as the mean ± SEM (n = 6 mice/group). *p< 0.05, ** *p* < 0.01, ***p < 0.001, treatment groups versus the NS control.

Reduced food intake and weight loss are the most prominent clinical features of cancer cachexia. As shown in Fig 4A, food intake every 48 h gradually decreased in MKN-45 xenograft mice. However, compared with that in the NS control group, food intake in the rhGH-treated groups increased. The total food intake for 14 days in the rhGH-treated groups at 2, 10, and 50 IU/kg body weight increased by 10.8%, 8.38% and 7.73% respectively, compared with that in the NS control group (Fig 4B). Considering the reduced food intake, the body weight of MKN-45 xenograft mice in the NS control group tended to decrease with time. However, MKN-45 xenograft mice treated with rhGH showed a significant increase in body weight compared with that in the NS control group(Fig 4C).The tumor-free body weight of mice treated with 2, 10, and 50 IU/kg body weight rhGH after 14 days from the first administration increased by 13.1% (*p*<0.01), 17.7% (*p*<0.001) and 23.4% (*p*<0.001), respectively, compared with that in the NS control group(Fig 4D); this effect was dose-dependent. These data indicate that rhGH can increase the food intake and body weight of MKN-45 xenograft mice, which can help to improve their nutritional status. A higher rhGH dosage appeared to be better than a lower dosage for increasing body weight.

### Effect of rhGH on the NK cell activity of PBMCs in MKN-45 xenograft mice

**Fig 5:**
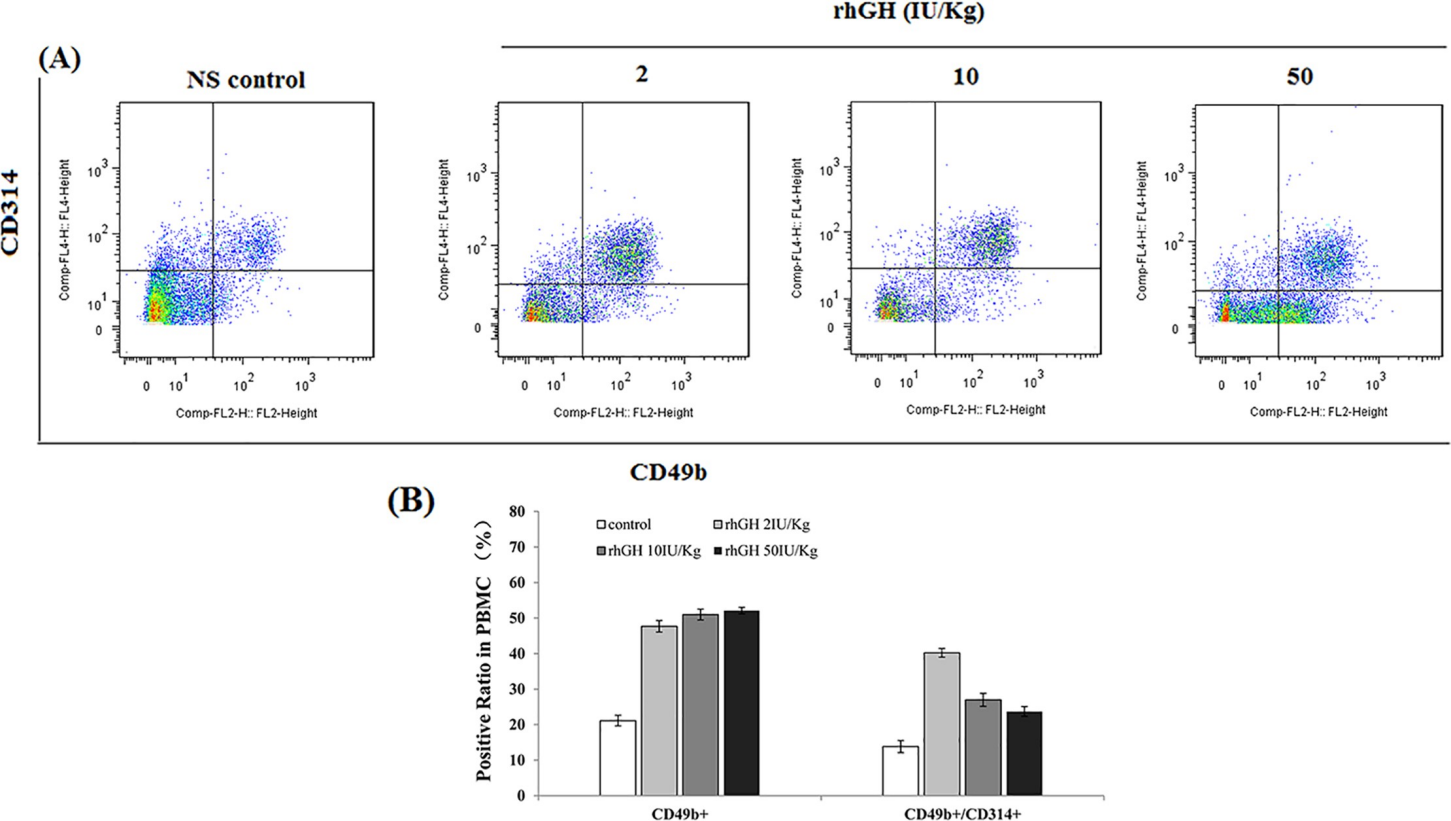
Effect of rhGH on NK activity of PBMCs in MKN-45 xenograft mice. (A) FACS analysis of the NK cell activity of PBMCs in MKN-45 xenograft mice. (B) Quantitative analysis of the mature NK cell percentage and active NK cell percentage of PBMCs. The data were obtained from three independent experiments and are presented as mean ± SEM. *p< 0.05, ** *p* < 0.01, treatment groups versus the control.

Due to the absence of the thymus in BALB/c nude mice, we selected NK cell activity as an indicator to evaluate the effect of rhGH on immune function in MKN-45 xenograft mice. As molecular markers on the NK cell surface, CD49b (DX-5) is a sign of mature NK cells, while CD314 (NKG2D) is a sign of active NK cells. As shown in Fig 5A, mature NK cells with CD49b-positive expression were detected in the upper right and lower right quadrants, and active NK cells with CD49b- and CD314-positive expression were detected in the upper right quadrant. The mature NK cell percentages in the PBMCs in the rhGH-treated groups(2, 10, and 50 IU/kg body weight) were significantly increased by 126.1% (*p*<0.01), 141.7% (*p*<0.01) and 146.9%(*p*<0.01), respectively, compared with those in the NS control group. At the same time, the active NK cell percentages of PBMCs in the rhGH-treated groups(2, 10, and 50 IU/kg body weight)were significantly increased by 191.3%(*p*<0.01), 95.7%(*p*<0.05) and 71.8%(*p*<0.05),respectively. This effect was negatively correlated with the dosage of rhGH, suggesting that rhGH had improved the immune function of MKN-45 xenograft mice and that a lower dosage appeared to be better than a higher dosage for increasing the active NK cell percentage.

## Discussion

In this study, we showed that tumor growth was not promoted by rhGH in MKN-45 xenograft mice, but the food intake was improved, the body weight was increased, and immune function was strengthened by treatment with rhGH.

Whether rhGH can promote tumor growth in vivo is a crucial factor for evaluating the safety of rhGH treatment in patients with advanced cancer. Our previous experiments in vitro had confirmed that rhGH showed no effect on MKN-45 cells because its GHR is negatively expressed. Yan L et al. [24] reported that the tumor volumes were not significantly different between the rhGH-treated groups and the control group throughout the entire treatment period. In the current study, we found that the growth of MKN-45 xenograft tumors was not affected by rhGH at middle and high dosages but was suppressed by rhGH at a low dosage compared with that of the control group. The differences in the results are likely due to the difference in the rhGH treatment dosage.

RhGH at a low dosage also down regulated the expression of Ki-67, VEGF and CD31 in MKN-45 xenograft tumors, a finding that was in accordance with its effect on MKN-45 xenograft tumor growth but with no effect on the expression of proteins involved in GHR-related signaling pathways. Thus, the suppression effect of low-dosage rhGH on MKN-45 xenograft tumors was not related to the GHR-related signaling pathways.

Food intake is considered a major indicator for evaluating the quality of life in animals [27]. Reduced food intake was found in all groups, but there was a significant increase in the food intake of MKN-45 xenograft mice treated with rhGH compared with that of MKN-45 xenograft mice treated with normal saline. Thus, rhGH improved quality of life in MKN-45 xenograft mice.

Loss of body weight is a main characteristic of cancer cachexia. When weight loss exceeds 30% of body weight, death normally ensues[28]. Thus, increasing the body weight is an important indicator for evaluating the nutritional status of cancer patients. The results showed that the body weight of all rhGH-treated groups significantly increased compared with that of the control group, especially in the high-dosage group, indicating that rhGH was beneficial for improving the nutritional status of MKN-45xenograft mice.

In addition to the nutritional status, immune function is also important in cancer and cancer cachexia treatment. FACS analysis of the NK cell activity of PBMCs in MKN-45 xenograft mice showed that rhGH treatment activated NK cells. In contrast to its effect on increasing body weight, the effect of rhGH on activating NK cells demonstrated a negative relationship with dosage. Thus, rhGH treatment at a low dosage achieved the best result in activating the NK cells of PBMCs in MKN-45 xenograft mice, likely related to the suppression effect on tumor growth induced by rhGH treatment at a low dosage.

Furthermore, the data of this article also suggest that the dosage of rhGH may be a factor worth considering if rhGH is used to cancer patients without GHR expression. Although treatment at a higher dosage achieved better results in increasing body weight, a lower dosage was more effective at strengthening immune function and suppressing the tumor growth in MKN-45 xenograft mice.

## Conclusion

RhGH was found to improve food intake, increase body weight and strengthen the immune function of MKN-45 xenograft mice but did not promote tumor growth. These data indicate that rhGH treatment may be beneficial for MKN-45 xenograft mice without risk of promoting tumor growth. MKN-45 xenograft mice treated with rhGH at a higher dosage gained more weight, while those treated with rhGH at a lower dosage showed stronger immune function and a smaller tumor volume.
